# Things Said of Us

**Published:** 1858-07

**Authors:** 


### THINGS SAID OF US.

"I AM a clergyman, and believe that true religion covers the whole ground of our being, body as well as soul. I lament the reckless violation of the laws of health and life, which I see daily. I wish to understand them more thoroughly, that I may instruct the ignorant; and want your JOURNAL as an additional teacher."

Another one writes, March 23d, 1858: "It is astonishing how much the papers have stolen out of your Journals, though some of them have been honest enough to credit you for them."

One of our religious exchanges says: "Dr. HALL'S JOURNAL OF HEALTH has an article on 'Religious Newspapers,' in which their virtue is magnified, perhaps, somewhat more than they deserve; and in which the Doctor gives the following confession of his exceedingly low estimate of human fame. He says:

"'We had rather be the writer of an eight or ten-lined paragraph, thought worthy of being quoted in the *American Messenger*, or the *Illustrated Christian Almanac*, with their half million readers, than to be the author of any volume ever published by the "Great Unknown," or the immortal "Boz," a paragraph for enduring good, not for the glittering glory of an hour, and as false as it is fair.

"We hope the above paragraph will be copied into the *American Messenger* or the *Illustrated Christian Almanac*, in order that the Doctor may, for once, attain his high ideal of literary satisfaction."

Our confrere seems to value human fame more, and religious newspaper influence less, than we do, and evidently thinks that we have a poor ambition. But we stand by our choice. Both before and since we wrote as above, various paragraphs of ours have appeared in the publications named; the one which provoked the criticism *was* copied, and in very many other papers also, showing that their editors accorded with our views.

Several religious newspapers have a circulation of from fifteen to thirty thousand; the *American Messenger* has about two hundred thousand distributed every month. One of these newspapers reaches twenty thousand families every week, and if edited in that catholic, liberal, and pious spirit which pervades every number of the *American Messenger*, it is impossible to estimate the influence for good which would be exerted by it. Very few of our ablest clergymen minister to the spiritual wants of a thousand families, and yet, one religious newspaper is the teacher and guide of twenty times the number. But these papers are read in families, which average four persons each. Is it a small ambition to desire to write a few lines, which may thrill the hearts of a hundred thousand people, with intellectual and pious emotion, which may wake them up to contemplations and activities which will happify themselves, and exalt and bless others! Does novel-reading do this? Are the people who read romances, any more than the writers of them, remarkable for their humanities, or for personal religion? We think not. The editors ought to know, that outside of Bible truth, there is no solid comfort, no solid happiness. WALTER SCOTT knew it; and one of his last recorded utterances was, to enjoin LOCKHART, his son-in-law, *to read the Bible, as the best and safest guide for man.*

No man in New York, perhaps, has labored more earnestly, efficiently, and boldly, for the better observance of the Sabbath day, than one of the editors of the paper we have named; the fiercest invectives we have almost ever heard, came from his lips, against the holders of the stocks and bonds of the railroads which were habitually operated on the Sabbath day; and yet the fame of "Boz" is coveted, the avowed champion of Sabbath desecration, against the better voice of his Queen and country! "Boz," the amateur play-actor—the man who has more than once left his country, to avoid paying his wine-merchant, his butcher, and his milkman. What line has SCOTT or DICKENS ever wrote, which will give safe and solid comfort in the fierce troubles of life, and in a dying hour? Not one.

No, no; we court not the fame of these men, their works, nor their practices; but we do covet the ability and the opportunity—the privilege—of being able to write, now and then, a sentence or two which will meet a million eyes, and go down into a million hearts, and wake up sympathies there, which will vibrate for a pure good, to the last hour of recorded time. And we are well assured that the *habitual feeling* of the three editors of the "*Independent*" is with us; and that the spirit in which the criticism was written was a transient intruder only.

Another exchange, from the Quaker City—a paper venerable by age and able in conduct, speaks well of our JOURNAL, then "buts" it all down, by saying: "His mission, however, is hardly that of setting the entire world to rights, as he sometimes seems to imagine."

So far from cherishing any such vain imagination, we have long since settled down in the conviction of an utter inability to set ourself right. The more we try, the more we don't succeed, apparently. But on the principle, that the more we try to help others, the more we do towards helping ourselves, we will aim high, although we have no expectation of reaching the mark, and one of our efforts will be, as heretofore, to induce the editors of the Religious Press to cultivate at all times, and under all circumstances, a high standard of gentlemanly forbearance, and Christian courtesy towards those who entertain different views from themselves. That there is a most discreditable lack of these amenities on the part of some of these gentlemen, is the painful observation of high-minded men. This glaring defect, we formerly explained, arises more from a bad digestion, than a bad heart. Were these strictures retaliatory?

